# Global Trends in Risk Factors for Low Back Pain: An Analysis of the Global Burden of Disease Study Data From 1990 to 2021

**DOI:** 10.1002/acr.25520

**Published:** 2025-04-03

**Authors:** Katharine E. Roberts, Manuela L. Ferreira, Paula R. Beckenkamp, Sneha Nicholson, Lyn March, Paulo H. Ferreira

**Affiliations:** ^1^ The University of Sydney Sydney New South Wales Australia; ^2^ The George Institute for Global Health Sydney New South Wales Australia; ^3^ The University of Washington Seattle; ^4^ The Kolling Institute Sydney New South Wales Australia

## Abstract

**Objective:**

The increasing burden associated with low back pain (LBP) is a critical issue. This is a novel analysis of trends in risk factors for LBP aiming to identify risk factors that require further attention or consideration in global policies to reduce the burden of LBP.

**Methods:**

The Global Burden of Disease study metadata were used to describe the trends in three modifiable categories of risk factors that contribute to the burden associated with LBP. The trends in occupational/ergonomic, behavioral (smoking), and metabolic (high body mass index [BMI]) risk factors for LBP between 1990 and 2021 have been described with attention to global areas, high sociodemographic index (SDI) areas, and low SDI areas.

**Results:**

The number of years lived with disability (YLDs) caused by LBP increased globally, in high and low SDI areas between 1990 and 2021. The impact of smoking and occupational/ergonomic risk factors have decreased; however, the impact of high BMI has increased markedly in the same time frame, with a particularly concerning impact in high SDI areas and on women.

**Conclusion:**

The burden of LBP is increasing globally, with a significant proportion of the YLDs caused by LBP attributed to three modifiable lifestyle factors: occupation/ergonomics, smoking, and high BMI. Of significant concern is the rapidly increasing impact of high BMI on YLDs caused by LBP, with the greatest impact seen among women in low and high SDI areas. The role of additional risk factors (eg, physical inactivity) still needs to be determined in the context of the global burden of LBP.

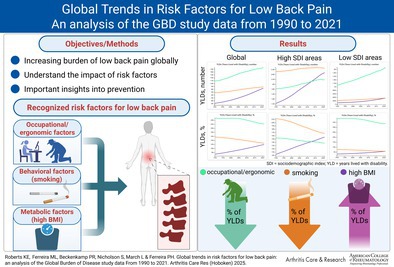

## INTRODUCTION

The increasing burden associated with low back pain (LBP) is a critical health issue that demands global attention. In 2020, 619 million people experienced LBP globally,^1^ with an age‐standardized prevalence of 7,460 per 100,000 (range 6,690–8,370), resulting in 69 million years lived with disability (YLDs) worldwide.^1^ This represents a significant increase from 1990, when 377.5 million people experienced LBP globally^2^ and the associated YLD was 43 million.^1^ Driving the demand for attention is the fact that LBP prevalence is forecast to expand to 843 (95% uncertainty interval [UI] 759–933) million by 2050, with the potential for substantial increases in associated disability and burden.^1^

**SIGNIFICANCE & INNOVATIONS**

- Understanding the trajectories of risk factors provides insights into preventive strategies that may positively impact low back pain (LBP) burden.
- The impact of occupational/ergonomic risk factors and smoking on the years lived with disability because of LBP is decreasing globally.
- The impact of high body mass index is increasing at an alarming rate and is particularly significant for women in both high sociodemographic index (SDI) areas and low SDI areas.
- We urgently need effective preventive strategies to address obesity, especially among women, if we aim to control the global burden of LBP.

Understanding the mechanisms that explain disease development and analyzing the trajectories of risk factors over time could provide insights into preventive strategies to positively impact disease prevalence and burden as well as identify areas that require further attention or a review in global policy.^3^ The Global Burden of Disease (GBD) study generates publicly available datasets and tools that allow the analysis of estimates of global health data. Specifically, GBD study data provide not only estimates of the prevalence of diseases but also the prevalence and impact of risk factors for them, affording the ability to identify the relative importance of different risk factors both over time and among populations globally.^3^

In 2018, *The Lancet* published an LBP series calling attention to the global dilemma of LBP,^4^, ^5^, ^6^ citing aging and expanding populations, as well as inadequate management, as major contributing factors associated with increasing disability and costs globally.^4^ In addition, the study demonstrated that in 2020 more than one‐third of the YLDs associated with LBP are associated with three GBD risk factors^1^: occupational/ergonomic, smoking, and high body mass index (BMI). These risk factors have been included in the GBD study LBP estimates, reflecting their relative prevalence in the literature when they meet the evidence of risk‐outcome pair criteria.^3^ The increased prevalence of these risk factors since 1990 may have been associated with the increasing burden of LBP globally. However, the trends in the risk association between these factors and LBP is still to be ascertained.

The current study aimed, for the first time, to report the global trends in risk factors for LBP from 1990 to 2021. The trends in YLDs have been reported as numbers and percentages providing a comparison between men and women as well as a comparison between the trends in risk factors in high sociodemographic index (SDI) areas and low SDI areas. Understanding the trends of risk factors and their association with the burden of LBP over time could provide insights into preventive strategies to positively impact LBP prevalence and its associated burden as well as important information to guide policy to address the global burden of LBP.

## PATIENTS AND METHODS

### Data source

The methods used by the GBD study for collecting and calculating LBP and risk factor estimates are described in detail elsewhere.^7^ In short, the GBD study uses a large number of data sources to estimate illness, injury, morbidity, and attributable risk for 204 countries.^7^, ^8^ Prevalence data and disability weights are then used to calculate YLDs. For LBP, considered to be a nonfatal health outcome, there were 492 input sources from 204 countries and territories identified between 1990 and 2021. Input data sources can be found at https://ghdx.healthdata.org/gbd-2021/sources. The GBD study follows the Guidelines for Accurate and Transparent Health Estimates Reporting Statement.^9^ The GBD study systematically reviews and synthesizes data from multiple electronic sources,^7^ and a meta‐regression approach is used to synthesize the data extracted for each risk‐outcome pair as per the Preferred Reporting Items for Systematic Reviews and Meta‐analysis framework.^10^ The main analytical tools used for GBD 2021 are disease model meta‐regression 2.1, spatiotemporal Gaussian process regression, and meta‐regression—Bayesian, regularised, trimmed.^7^

### Outcome

The GBD study defines LBP as pain experienced in the posterior body between the lower margin of the 12th ribs and the lower gluteal folds, which may or may not include referred pain into one or both lower limbs, and lasts for at least 1 day.^1^, ^11^ YLDs were used to describe the burden associated with LBP. Although disability‐adjusted life years (DALYs) are often used to describe the burden associated with disease, YLDs are commonly used to describe the burden of nonfatal diseases such as LBP.^1^ Estimates are presented as numbers (count), age‐standardized percentages, and age‐standardized rates (per 100,000 population). The GBD study categorizes risk factors into hierarchical order. Level 1 represents three broad categories (occupational, behavioral, and metabolic), which are progressively broken down into level 2, 3, and 4 risk factors.^3^ For example, behavioral risk factors (level 1) may be disaggregated into tobacco (level 2) and smoking, chewing tobacco, and secondhand smoke (level 3). This hierarchy allows analysis of individual risk factors or groups of risk factors.^7^

### Risk factors

In the context of LBP, the risk factors reported in the GBD study are occupational/ergonomic (level 3), smoking (levels 2 and 3), and BMI (level 2).^3^ Occupational/ergonomic exposures include lifting, forceful movements, vibrations, and awkward postures. High BMI for adults aged >20 years is defined as >20 to 25 kg/m^2^.^12^ SDI is an indicator of the social and economic conditions (ie, development status) that influence health outcomes. SDI is calculated by the GBD combining total fertility rate, mean education, and lag‐distributed income per capita,^8^ generating a score between 0 and 1. A score of 0 represents minimum socioeconomic development and, therefore, poorer associated health outcomes.^7^ SDI is divided into quintiles with low SDI incorporating countries with an SDI of 0.00 to 0.45 and high SDI incorporating countries with an SDI of 0.81 to 1.00.^13^

The GBD study assesses the impact of each risk factor using a comparative risk assessment framework^7^ estimating risk with a six‐step meta‐analytical method: (1) risk‐outcome pairs that meet specific criteria; (2) relative risks as a function of exposure; (3) levels of exposure in each age, sex, location and year; (4) the theoretical minimum risk exposure; (5) computed attributable deaths, years of life lost, YLDs, and DALYs; and (6) population‐attributable fractions and attributable burden.^3^ The GBD study currently provides data on only the three risk factors discussed in this study, for which there is credible evidence of risk‐outcome relationships. This includes findings supported by more than one study type, data from at least two cohorts, minimal and explained heterogeneity, low risks of confounding and selection bias, and biologically plausible dose‐response gradients.^1^, ^7^

### Data presentation, UIs, and data access

All data were downloaded from the results^14^ and compare tools^15^ for presentation. The GBD metadata are publicly available through the Institute for Health Metrics and Evaluation (IHME) Global Health Data Exchange (GHDx) at https://www.healthdata.org/data-tools-practices/interactive-data-visuals and https://vizhub.healthdata.org/gbd-compare/.

Trends in risk factors were viewed using the IHME data visualization tools. Specifically, the GBD “results tool”^14^ was used to visualize the trends in risk factors for LBP using 5‐year brackets (1990 to 2020 and 2021). The GBD “compare tool”^15^ was used to visualize the risk factors for LBP when comparing high SDI countries with low SDI countries. Percentage change was generated by the data visualization tools. The visualization tools were accessed in July 2024.

No patients were involved in this study, and ethical approval was not required for this study. All data relevant to the study are included in the article or uploaded as online supplemental information. The GBD metadata are publicly available through the IHME GHDx.

## RESULTS

**Figure 1:**
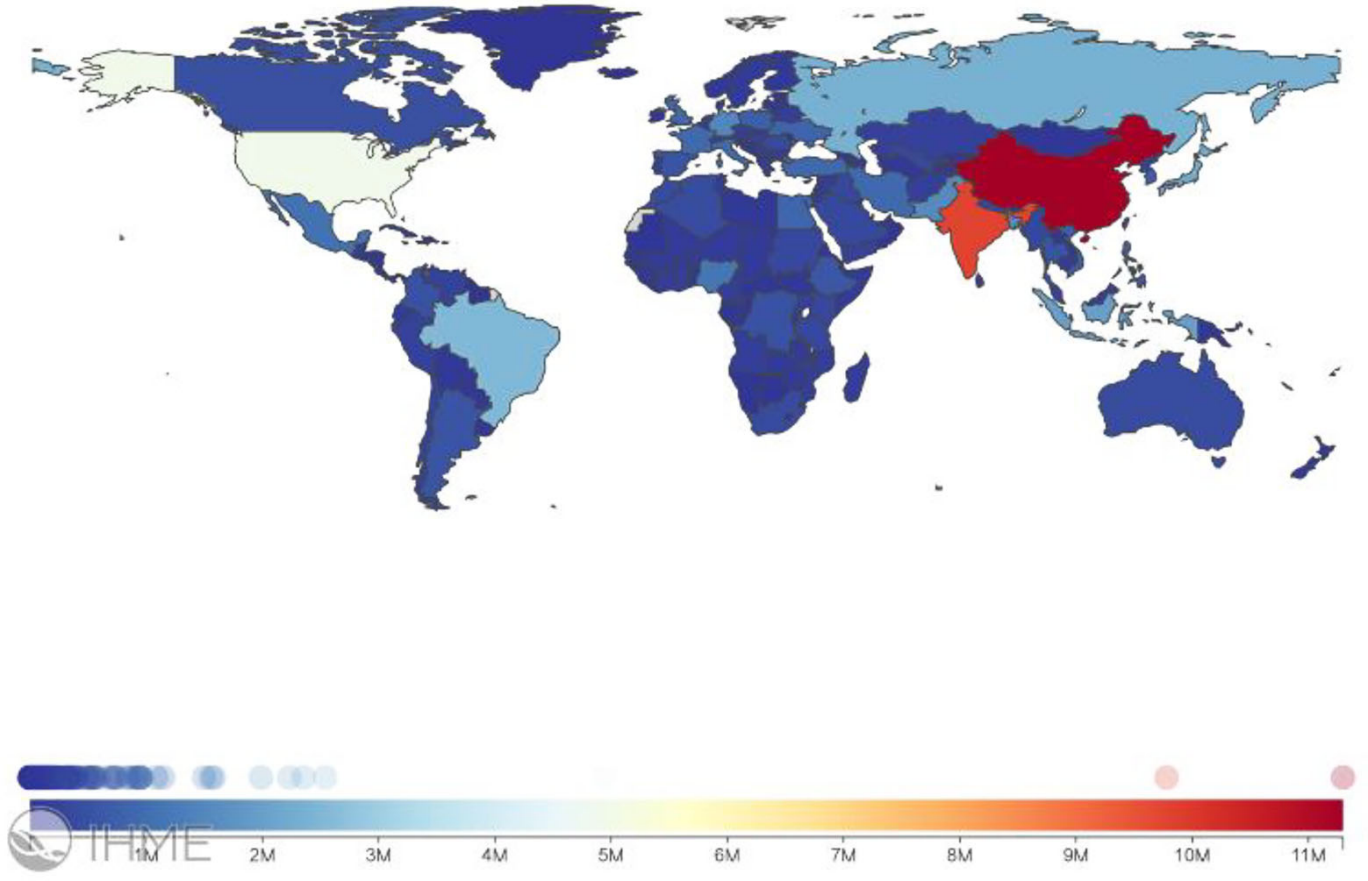
Global distribution of the estimated number of years lived with disability caused by low back pain in 2021 presented as all ages and both sexes.

In 1990, 42.4% of the YLDs caused by LBP globally were attributable to exposure to the three risk factors assessed in this study: occupational/ergonomic, behavioral (smoking), and metabolic (high BMI). By 2020, the proportion had decreased slightly to 38.8%.^1^ A global map displaying YLDs caused by LBP in 2021 is presented in Figure 1, and global maps displaying LBP attributable to occupational/ergonomic, behavioral (smoking), and metabolic (high BMI) risk factors for both sexes and all ages in 2021 are available in Supplementary Figures 1, 2, and 3, respectively.

### Global trends in YLDs attributable to occupational/ergonomic, smoking, and high BMI risk factors

**Figure 2:**
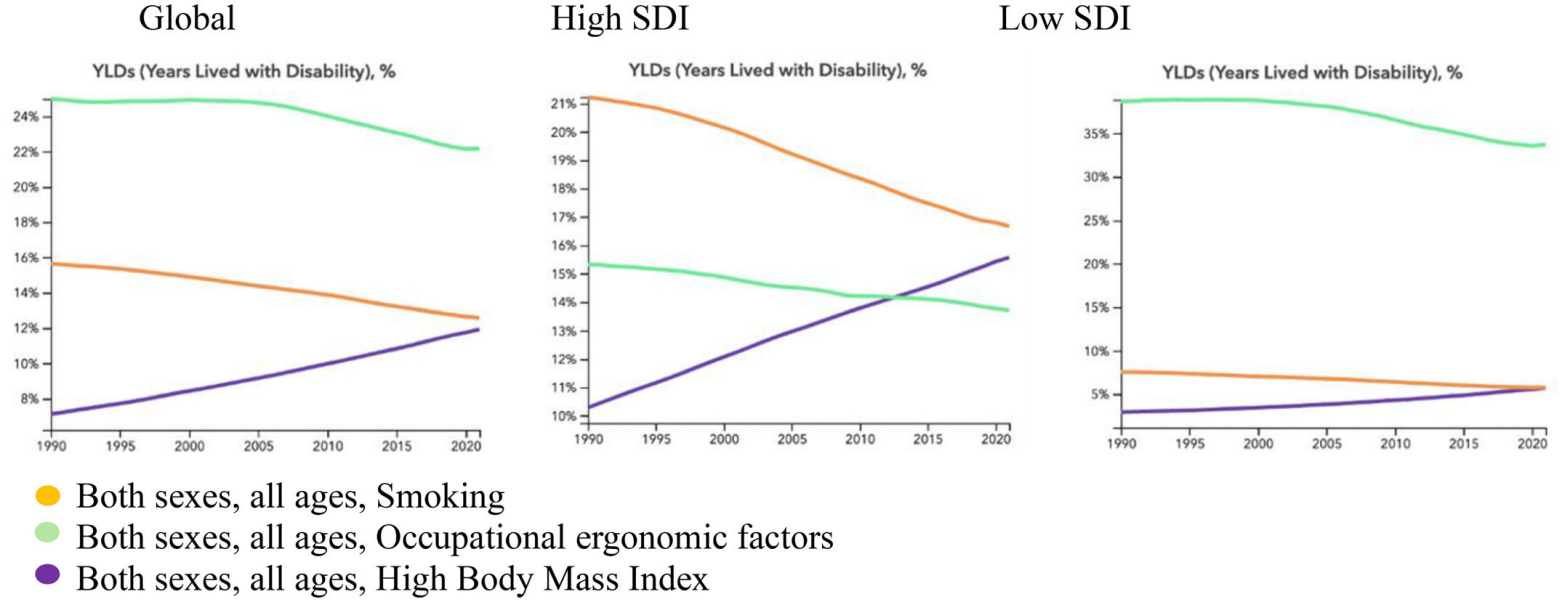
Estimated trends in YLDs caused by occupational/ergonomic, behavioral (smoking), and metabolic (high body mass index) risk factors presented as percentages from 1990 to 2021. SDI, Sociodemographic Index; YLD, year lived with disability. Color figure can be viewed in the online issue, which is available at http://onlinelibrary.wiley.com/doi/10.1002/acr.25520/abstract.

**Figure 3:**
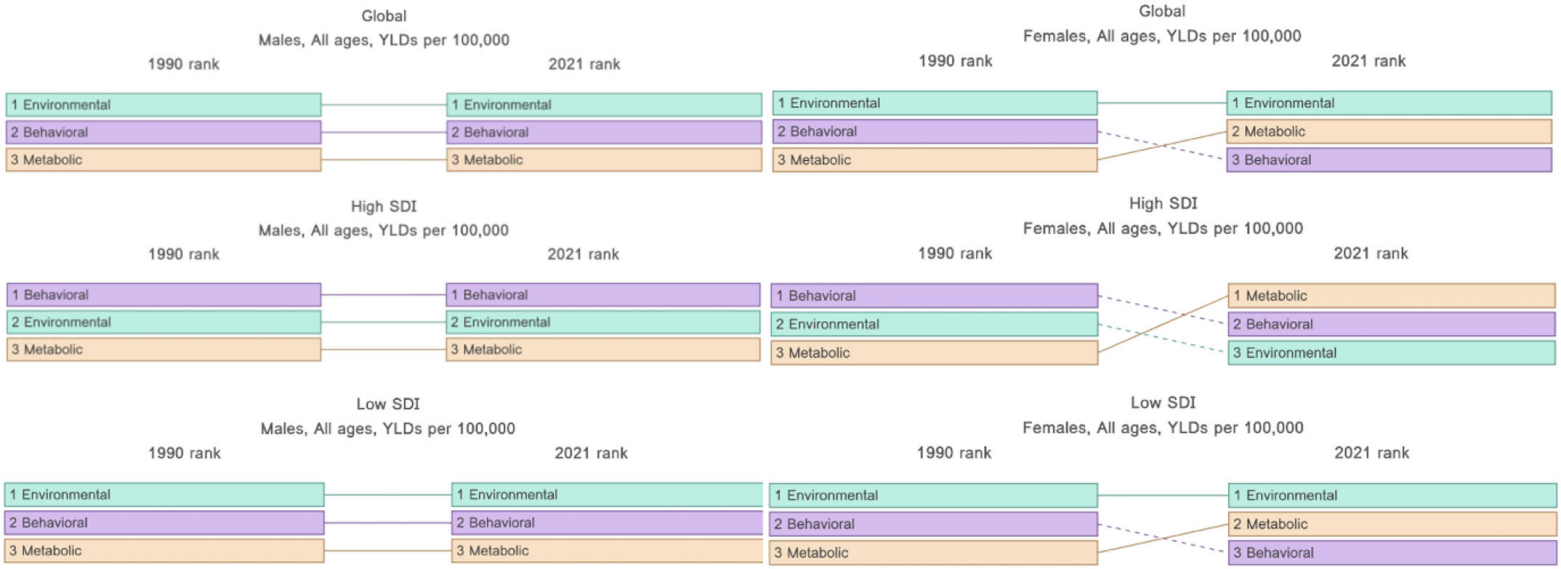
Rank of (occupational/ergonomic), behavioral (smoking), and metabolic (high body mass index) risk factors as an estimated rate of YLDs per 100,000 caused by low back pain in 1990 compared with 2021 for all Global Burden of Disease age groups. SDI, Sociodemographic Index; YLD, years lived with disability. Color figure can be viewed in the online issue, which is available at http://onlinelibrary.wiley.com/doi/10.1002/acr.25520/abstract.

Globally, the YLDs caused by LBP as a percentage of total YLDs attributable to all causes decreased from 1990 to 2021 for occupational/ergonomic and smoking risk factors, whereas the percentage of YLDs caused by LBP attributable to high BMI increased in the same period (Figure 2). The rank of risk factors by contribution to YLDs caused by LBP has occupational/ergonomic as the most contributing factor, followed by smoking and high BMI, with occupational/ergonomic factors contributing the most YLDs caused by LBP and high BMI contributing the least (Figure 3). That rank order has not changed since 1990.

**Figure 4:**
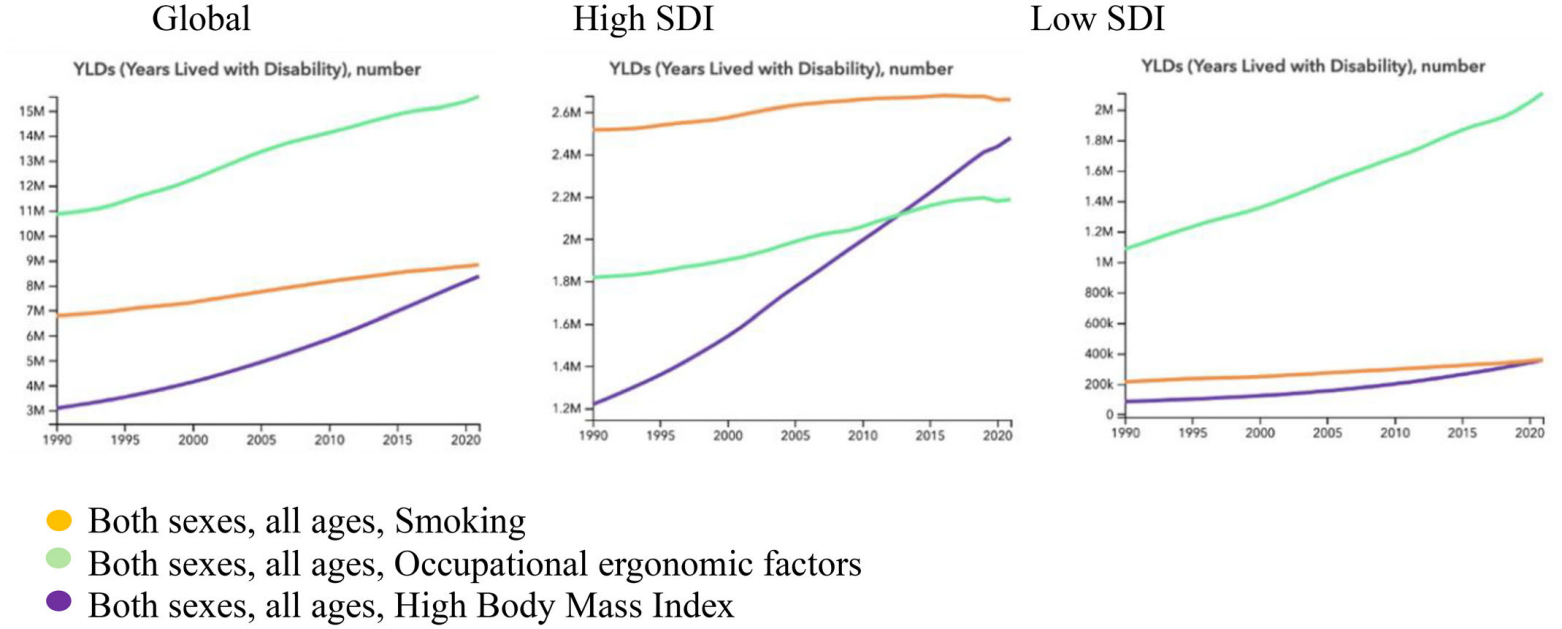
Estimated trends in YLDs caused by occupational/ergonomic, behavioral (smoking), and metabolic (high body mass index) risk factors presented as counts from 1990 to 2021. SDI, Sociodemographic Index; YLD, year lived with disability. Color figure can be viewed in the online issue, which is available at http://onlinelibrary.wiley.com/doi/10.1002/acr.25520/abstract.

The global rate of YLDs caused by LBP attributed to occupational/ergonomic risk factors has decreased for both sexes combined and when men and women are considered separately. The percentage of YLDs caused by LBP attributed to occupational/ergonomic risk factors and smoking as a proportion of total YLDs has also decreased (by 12% and 21%, respectively) from 1990 to 2021. In comparison, the percentage of YLDs caused by LBP attributed to high BMI as a proportion of total YLDs increased by 65% from 1990 to 2021. Importantly, the number of YLDs caused by exposure to high BMI also increased markedly from 3,100,000 in 1990 to 8,400,000 in 2021 (Table 1; Figure 4; and Supplementary Figures 4 and 5).

**Table 1:** Percentage change in YLDs caused by LBP/total YLDs, number of YLDs, and rate of YLDs per 100,000 attributed to occupational, smoking, and high BMI risk factors for men, women, and both sexes combined between 1990 and 2021*

Location	Risk factor	Men, %^a^	Women, %^a^	Both sexes, %^a^
Percentage change in the number of YLDs (all ages)				
Global	Behavioral (smoking)	35 (29 to 40)	22 (16 to 27)	30 (25 to 35)
Global	Metabolic (high BMI)	178 (160 to 194)	167 (154 to 179)	171 (157 to 183)
Global	Occupational/ergonomic	37 (32 to 43)	50 (40 to 61)	43 (37 to 50)
High SDI	Behavioral (smoking)	8 (2 to 14)	3 (−3 to 8)	6 (0 to 11)
High SDI	Metabolic (high BMI)	116 (98 to 134)	96 (83 to 111)	103 (88 to 119)
High SDI	Occupational/ergonomic	18 (12 to 24)	23 (14 to 33)	20 (14 to 27)
Low SDI	Behavioral (smoking)	71 (63 to 77)	66 (50 to 85)	69 (62 to 77)
Low SDI	Metabolic (high BMI)	401 (320 to 452)	308 (273 to 334)	333 (289 to 360)
Low SDI	Occupational/ergonomic	86 (79 to 94)	102 (91 to 114)	95 (88 to 102)
Percentage change in the YLDs owing to LBP/total YLDs (age‐standardized)				
Global	Behavioral (smoking)	−21 (−24 to −20)	−31 (−34 to −29)	−25 (−27 to −23)
Global	Metabolic (high BMI)	65 (54 to 73)	53 (46 to 60)	57 (48 to 63)
Global	Occupational/ergonomic	−12 (−15 to −9)	−6 (−11 to 0.5)	−9 (−12 to −5)
High SDI	Behavioral (smoking)	−25 (−28 to −22)	−24 (−27 to −21)	−24 (−26 to −21)
High SDI	Metabolic (high BMI)	53 (40 to 63)	46 (37 to 56)	48 (37 to 58)
High SDI	Occupational/ergonomic	−8 (−11 to −5)	0.1 (−6 to 7)	−4 (−7 to 0)
Low SDI	Behavioral (smoking)	−21 (−24 to −18)	−24 (−32 to −16)	−23 (−26 to −20)
Low SDI	Metabolic (high BMI)	129 (92 to 154)	77 (63 to 88)	92 (73 to 102)
Low SDI	Occupational/ergonomic	−15 (−18 to −12)	−12 (−17 to −7)	−14 (−17 to −10)
Percentage change in the rate of YLDs per 100,000 (age‐standardized)				
Global	Behavioral (smoking)	−31 (−33 to −29)	−39 (−41 to −36)	−33 (−35 to −32)
Global	Metabolic (high BMI)	45 (36 to 53)	37 (30 to 43)	39 (32 to 45)
Global	Occupational/ergonomic	−23 (−25 to −20)	−16 (−21 to −10)	−19 (−22 to −16)
High SDI	Behavioral (smoking)	−30 (−36 to −27)	−30 (−33 to −27)	−29 (−32 to −26)
High SDI	Metabolic (high BMI)	41 (29 to 52)	35 (26 to 45)	36 (26 to 46)
High SDI	Occupational/ergonomic	−15 (−18 to −11)	−8 (−13 to −1)	−11 (−15 to −8)
Low SDI	Behavioral (smoking)	−26 (−29 to −23)	−29 (−36 to −21)	−27 (−31 to −24)
Low SDI	Metabolic (high BMI)	114 (80 to 137)	66 (53 to 78)	81 (63 to 92)
Low SDI	Occupational/ergonomic	−21 (−24 to −18)	−17 (−22 to −12)	−19 (−22 to −16)

* BMI, body mass index; LBP, low back pain; SDI, sociodemographic index; YLD, year lived with disability.

^a^ The data in parentheses are uncertainty intervals.

The impact of smoking on LBP is more pronounced for men than it is for women globally. The total number of YLDs caused by LBP for men that were attributable to smoking increased from approximately 4,400,000 in 1990 to 5,900,000 in 2021 (Supplementary Figure 4). In comparison, the YLDs caused by LBP that were attributable to smoking for women increased from approximately 2,400,000 in 1990 to 2,900,000 in 2021 (Supplementary Figure 4). Most notably, the impact of high BMI on YLDs caused by LBP has increased globally since 1990. The rate of YLDs per 100,000 caused by LBP attributed to high BMI for men increased by 45% from 1990 to 2021. This trend is also significant for women in the same period, for whom the rate of YLDs per 100,000 caused by LBP because of high BMI increased by 37% (Table 1 and Supplementary Figure 4).

### Trends in YLDs attributable to occupational/ergonomic, smoking, and high BMI risk factors in high SDI areas

The trends in high SDI areas mimic those of global trends, with YLDs caused by LBP as a proportion of total YLDs decreasing for occupational/ergonomic and smoking risk factors and increasing for high BMI between 1990 and 2021 (Figure 2). In high SDI areas, when considering both sexes combined, the impact of high BMI increased by 36% from 118.84 YLDs per 100,000 in 1990 to 161.80 YLDs per 100,000 in 2021 (Table 2; Supplementary Figure 4). A slight 11% decrease occurred in rate of YLDs per 100,00 caused by occupational/ergonomic risk factors with a more significant decrease of 29% in rate of YLDs per 100,000 caused by smoking for both sexes combined in that timeframe. Notably, in high SDI areas, the rank order differs, and smoking is more impactful than occupational/ergonomic risk factors or high BMI for both sexes combined. This indicates that smoking has a bigger impact on the risk of YLDs caused by LBP in high SDI areas than low SDI areas or globally when considering both sexes combined. In 2012/2013, the impact of high BMI surpassed that of occupational/ergonomic factors and the rank order changed, with smoking as the most impactful risk factor followed by high BMI and then occupational/ergonomic risk factors. This trend in the ranking of risk factors remained stable from 2013 to 2021 (Table 1 and Supplementary Figure 4).

**Table 2:** Years lived with disability caused by low back pain presented as counts (millions), percentage (age‐standardized), and rate (age‐standardized) for global, high SDI, and low SDI areas attributed to occupational, behavioral (smoking), and metabolic (high BMI) risk factors in 1990 and 2021 (both sexes combined)*

Risk	Metric^a^	1990^b^	2021^b^
Global			
Metabolic (high BMI)	Number	3.09 (0.31–6.48)	8.36 (0.84–17.42)
Metabolic (high BMI)	Percent	7.49 (0.79–14.72)	11.74 (01.22–22.84)
Metabolic (high BMI)	Rate	70.22 (7.14–146.48)	97.66 (9.78–204.00)
Occupational/ergonomic	Number	10.85 (7.60–14.54)	1.57 (11.03–20.91)
Occupational/ergonomic	Percent	24.18 (22.35–25.88)	22.07 (20.34–23.69)
Occupational/ergonomic	Rate	226.82 (158.99–304.34)	183.82 (129.49–247.24)
Behavioral (smoking)	Number	6.78 (4.07–10.07)	8.82 (5.18–13.13)
Behavioral (smoking)	Percent	16.34 (11.13–21.32)	12.26 (8.16–16.33)
Behavioral (smoking)	Rate	153.22 (91.37–226.59)	102.04 (60.03–152.10)
High SDI			
Metabolic (high BMI)	Number	1.22 (0.12–2.55)	2.48 (0.25–5.09)
Metabolic (high BMI)	Percent	9.99 (1.03–19.78)	14.80 (1.55–28.27)
Metabolic (high BMI)	Rate	118.84 (11.60–248.80)	161.80 (15.99–332.59)
Occupational/ergonomic	Number	1.82 (1.27–2.45)	2.19 (1.56–2.95)
Occupational/ergonomic	Percent	15.68 (14.47–16.82)	15.12799 (14.10–16.23)
Occupational/ergonomic	Rate	186.74 (129.62–252.22)	165.69 (118.62–222.72)
Behavioral (smoking)	Number	2.52 (1.50–3.74)	2.66 (1.56–3.99)
Behavioral (smoking)	Percent	20.77 (14.02–27.24)	15.88 (10.35–21.31)
Behavioral (smoking)	Rate	247.25 (147.56–367.62)	173.83 (101.74–261.13)
Low SDI			
Metabolic (high BMI)	Number	0.08 (0.01–0.17)	0.36 (0.04–0.73)
Metabolic (high BMI)	Percent	3.22 (0.36–6.10)	6.16 (0.62–11.91)
Metabolic (high BMI)	Rate	27.81 (3.09–55.76)	50.30 (5.07–103.18)
Occupational/ergonomic	Number	1.08 (0.77–1.45)	2.11 (1.47–2.84)
Occupational/ergonomic	Percent	38.38 (35.72–40.91)	33.04 (30.52–35.33)
Occupational/ergonomic	Rate	331.67 (238.02–442.90)	269.53 (191.97–364.56)
Behavioral (smoking)	Number	0.21 (0.12–0.32)	0.36 (0.20–0.55)
Behavioral (smoking)	Percent	8.69 (5.70–11.74)	6.69 (4.29–9.08)
Behavioral (smoking)	Rate	75.09 (44.42–112.87)	54.56 (31.26–83.63)

* BMI, body mass index; SDI, sociodemographic index.

^a^ Number is presented as counts in millions for all ages. Percentages and rates are age‐standardized.

^b^ The data in parentheses are uncertainty intervals.

When considering men alone, a decrease is observed of 8% and 25% of total YLDs caused by exposure to occupational/ergonomic risk factors and smoking, respectively, and an increase of 53% of total YLDs caused by exposure to high BMI between 1990 and 2021. In comparison, the YLDs caused by LBP attributed to smoking in women decreased by 24% and only 0.1% for occupational/ergonomic risk factors between 1990 and 2021. The rate of YLDs per 100,000 caused by LBP attributed to high BMI has increased from 95.48 in 1990 to 134.88 in 2021, representing an increase of 35% in total YLDs. Of note, the rank order of risk factors for women has changed significantly, with high BMI becoming more impactful throughout the period from 1990 to 2021 (Figure 3). In 1990, smoking was the most impactful risk factor for women followed by occupational/ergonomic risk factors then high BMI. In 1996, high BMI outranked occupational risk factors, and by 2012 high BMI became the most impactful risk factor for YLDs caused by LBP in women in high SDI areas.

### Trends in YLDs attributable to occupational/ergonomic, smoking, and high BMI risk factors in low SDI areas

In low SDI areas, the ranks mirror those of the global trends for both sexes combined (Figure 3). However, when women are considered separately, high BMI becomes more impactful than behavioral risk factors from 1991 to 2021 (Supplementary Figure 6). Similar to the global and high SDI area trends, the number of YLDs caused by LBP attributable to occupational/ergonomic risk factors, smoking, and high BMI increased from 1990 to 2021. However, in contrast to global and high SDI trends, the percentage of YLDs attributable to occupational/ergonomic risk factors did not start to decline until 2005, and the percentage of YLDs attributable to high BMI demonstrated a slow increase from 1990 to 2021 (Figure 2).

The impact of risk factors in low SDI areas for both sexes combined have the same rank order as the global trends: occupational risk factors are ranked first followed by smoking and high BMI (Figure 3). In low SDI areas, occupational/ergonomic risk factors have been the most impactful risk factors associated with YLDs caused by LBP since 1990, declining by a minimal 14% of total YLDs by 2021 (Table 1). The rate of YLDs per 100,000 caused by LBP attributable to occupational risk factors also decreased slightly for both sexes combined. Of note, YLDs caused by LBP attributed to exposure to smoking and high BMI increased throughout the period, resulting in 3,600,000 total YLDs caused by LBP in 2021 for each risk factor. Despite this increase in total YLDs, the impact of smoking as a proportion of total YLDs decreased by 23% when considering both sexes combined.

Of particular significance, the impact of high BMI as a proportion of total YLDs increased by 92% by 2021 in both sexes combined. Considering women alone, a decrease is seen in the impact of occupational/ergonomic risk factors and smoking of 12% and 24% of total YLDs caused by LBP as a proportion of total YLDs, respectively. The impact of occupational/ergonomic risk factors and smoking on the rate of YLDs per 100,000 similarly decreased by 17% and 29%, respectively. The impact of high BMI on all measures of YLDs caused by LBP increased markedly. Most notably, the number of YLDs caused by LBP that are attributed to high BMI increased by a staggering 308% by 2021 (Table 1 and Supplementary Figure 4).

## DISCUSSION

This descriptive study aimed to explore the trends in trajectories of three modifiable risk factors for LBP (occupational/ergonomic, behavioral [smoking], and metabolic [high BMI]) from 1990 to 2021, using data from the IHME and GBD study. Significant changes were observed over time of the impact of these risk factors on the total YLDs caused by LBP, the rate of YLDs per 100,000, the YLDs caused by LBP as a proportion of total YLDs owing to all causes, and, importantly, the rank order of these risk factors.

Although occupational/ergonomic risk factors are the most impactful risk factors for LBP globally and in low SDI areas, a decline in the percentage of YLDs owing to occupational/ergonomic risk factors has been seen worldwide. Of note, this decline was not observed in low SDI areas until the early 2000s. In addition, a decrease in average annual percent change in DALY rates and age‐standardized disability rates since 1990 that has been seen in high SDI areas has not been seen in low SDI areas,^16^ suggesting that low SDI areas have been slower to respond to the burden of musculoskeletal disorders,^16^ potentially explaining this delay.

In high SDI areas, the number of YLDs that can be attributed to smoking is a decreasing trend that has been occurring since 2011, and the percentage of YLDs caused by LBP as a proportion of total YLDs and the rate of YLDs per 100,000 that can be attributed to smoking has decreased globally since 1990, demonstrating the success that global policy can have in modifying behavior. The World Health Organization Framework Convention on Tobacco Control was enforced in 2005,^17^ with a large number of countries globally experiencing their most significant reductions in age‐standardized prevalence of smoking between 2005 and 2009^17^ despite disparities still existing in global taxation and legislation.^3^ A positive correlation between smoking and SDI has been reported,^12^, ^18^ which may reflect the significant use of smokeless tobacco in low SDI areas^18^ and the ongoing challenge of changing lifestyle behaviors globally.^18^ Furthermore, increasing global populations and population ageing^18^ are resulting in increased numbers of people smoking and increased YLDs owing to LBP attributed to smoking, despite a decrease in the global prevalence of smoking.^17^

The most concerning trend identified is the rapidly increasing impact of high BMI globally on YLDs caused by LBP with an increase of 171% globally, 103% in high SDI areas and 333% in low SDI areas. Globally, the DALYs attributed to high BMI increased from 33.1 million in 1990 to 70.7 million in 2017,^19^ which is likely because of increasing and aging populations^19^ as well as increasingly sedentary lifestyles, increased caloric intake,^3^ and increased life‐stress.^19^ The risk‐weighted exposure for high BMI is noted to increase with increasing SDI,^20^ with summary exposure values increasing by >40% between 1990 and 2016.^18^ The rapidly increasing impact of high BMI on YLDs caused by LBP in high SDI areas is particularly worrisome as all‐cause DALYs attributable to high BMI have remained stable in high SDI areas over this same period of time.^21^

In 2021, the impact of metabolic risk factors on YLDs caused by LBP was markedly more significant for women than for men, with the discrepancy likely to be multifactorial. Obesity is more prevalent in women than men globally^22^, ^23^ and is more common in older age groups.^22^ Women may be more susceptible to psychopathology associated with obesity and are up to two times more likely to report anxiety or affective disorders associated with obesity,^24^ as well as decreased health‐related quality of life,^25^ compared with men. Increased abdominal and visceral fat associated with hormonal changes that occur during perimenopause and menopause may also be important in this relationship,^26^ as chronic low‐grade inflammation is specifically associated with abdominal and visceral adiposity.^27^ Furthermore, low levels of physical activity have been found to be associated with an increased incidence of radiating LBP in people with obesity (odds ratio [OR] 3.3, 95% confidence interval [CI]1.01–10.4).^28^ In addition, the bidirectional relationship among high BMI, low physical activity, and LBP is likely to be important.

Increased awareness and intervention to mitigate risks, as well as technology and automation and a shift away from manufacturing and agriculture, has likely driven the decreasing impact of occupational/ergonomic risk factors on YLDs caused by LBP.^20^ Globalization has led to increased manufacturing in lower SDI areas, which may have decreased the impact of occupational risk factors on YLDs caused by LBP in high SDI areas while increasing the impact in low SDI areas. In high SDI areas, fewer YLDs caused by LBP can be attributed to occupational/ergonomic risk factors than to smoking.

Low physical activity is almost certainly an important risk factor that is contributing to the increase in YLDs caused by LBP globally, and it would be appropriate for the IHME to consider inclusion of low physical activity as a risk factor for musculoskeletal conditions, including LBP, in future GBD releases. There is global recognition of an increasing epidemic of sedentary behavior partly associated with changing occupational and leisure time activities.^29^, ^30^ Alzrahani et al performed two systematic reviews reporting an inverse relationship between physical activity and LBP, with moderate physical activity associated with lower LBP prevalence,^31^ and >3 hours per day of sedentary behavior associated with increased LBP disability.^32^ Psychological state is also recognized as a potential risk factor for LBP,^33^, ^34^, ^35^ and a history of LBP may be associated with recurrences of LBP,^36^ both of which warrant further attention on a global scale.

Although the impact of occupational/ergonomic risk factors on LBP is a decreasing trend, it warrants continued attention, especially in low SDI areas. Lifting, forceful movements, vibrations, and awkward postures are most commonly associated with manufacturing, farming, and manual labor,^37^, ^38^ however it is still unclear whether LBP is more prevalent in rural or urban settings.^39^ Recent evidence also suggests psychosocial aspects, such as high stress levels,^40^, ^41^, ^42^ may also be important contributors to LBP associated with occupational/ergonomic risk factors.^39^

The mechanisms through which high BMI impacts LBP are multifactorial with systemic inflammation,^43^, ^44^ increased mechanical stress,^45^, ^46^ metabolic syndrome,^45^ and deconditioning associated with low physical activity^47^, ^48^ thought to contribute to the development of LBP. People with a high BMI commonly report higher disability levels associated with their LBP,^49^ with the odds of experiencing high levels of LBP disability associated with high BMI being 1.39 (95% CI 1.15–1.68) in men and 1.45 (95% CI 1.29–1.62) in women.^49^ Moreover, men and women who are obese are more likely to seek medical care for their LBP (OR 1.56, 95% CI 1.46–1.67)^50^ compared with people with a healthier BMI. The impact of high BMI on the chronicity of LBP, disability associated with LBP, and care‐seeking for LBP^49^, ^50^, ^51^, ^52^ suggests that lifestyle programs focusing on maintaining a healthy BMI could potentially be important factors in managing the global burden associated with LBP.^53^

There appears to be a bidirectional relationship between smoking and LBP,^54^ with smoking impacting LBP via central processing,^54^ altered pain processing, and impaired oxygen delivery^55^ with further possible links via low mood, socioeconomic status, and opioid use.^55^ A recent meta‐analysis reported that current smokers are 30% more likely to suffer from chronic disabling LBP than nonsmokers.^56^ Smokers with chronic pain also report increased pain intensity,^54^, ^57^ poorer function, worse mental health and mood,^54^ worse pain interference,^54^ and greater opioid use^57^ compared with nonsmokers.^54^, ^58^ Smoking cessation is likely to improve musculoskeletal health,^59^ day‐to‐day functional capacity,^60^ and the incidence of LBP.^61^

The LBP series published in *The Lancet* ^4^, ^5^, ^6^ made a call for LBP management to focus on healthy lifestyles, suggesting that lifestyle programs that focus on these modifiable lifestyle behaviors may be important for managing the global burden associated with LBP. Understanding the trends in the trajectories of these modifiable lifestyle risk factors for LBP may also provide important information for guiding global conversation and policy to address the burden associated with LBP. Strategies may need to vary between areas of different SDIs, addressing differences in population and access to resources as well as society and culture.^6^ Understanding the impact and trends of these lifestyle risk factors in different SDI areas may, therefore, aid in directing focus for more effective policy changes and priorities globally.

To our knowledge, this is the first study describing the trends in three modifiable risk factors for LBP from 1990 to 2021, which is a significant strength of this study. The large volume of data generated by the GBD study and the 29 years represented in the data are further strengths of this study. However, the data generated by the GBD study are modeled estimates, which may be seen as a limitation of this study.^62^ The LBP prevalence data are primarily obtained from high‐income countries, which may impact the generalizability of the results.^1^ However, we did not discuss subnational data or specific countries in an effort to maximize the completeness of data used in the trend visualizations. Furthermore, although temporal changes in GBD data are thought to be potentially unreliable,^62^ we focused on a general trend analysis rather than specific changes by year. Data from low SDI countries are scarce in the GBD analyses, impacting the strength of conclusions from low SDI areas,^63^ and conclusions should therefore be considered cautiously. Additionally, a large component of risk of LBP is unaccounted for in GBD data, and additional factors such as low physical activity, if measured, may influence the rank order of modifiable factors.

The burden of LBP continues to increase globally, with a significant portion of the YLDs caused by LBP attributed to three modifiable lifestyle factors: occupational/ergonomic, smoking, and high BMI. The number of YLDs caused by LBP continues to increase globally despite the percentage of total YLDs decreasing, which is reflective of an expanding and aging global population. Exposure to occupational risk factors remains the biggest contributor to YLDs caused by LBP globally, but of greater concern is the rapidly increasing impact of high BMI, especially among women. The global increase in BMI is likely related to increases in sedentary lifestyles, which may further impact YLDs caused by LBP. Local and global strategy and policy urgently need to consider the management of the modifiable lifestyle risk factors of occupational/ergonomic, behavioral (smoking), and especially metabolic (high BMI) risk factors to address the growing global burden associated with LBP.

## AUTHOR CONTRIBUTIONS

All authors contributed to at least one of the following manuscript preparation roles: conceptualization AND/OR methodology, software, investigation, formal analysis, data curation, visualization, and validation AND drafting or reviewing/editing the final draft. As corresponding author, Dr Roberts confirms that all authors have provided the final approval of the version to be published and takes responsibility for the affirmations regarding article submission (eg, not under consideration by another journal), the integrity of the data presented, and the statements regarding compliance with institutional review board/Declaration of Helsinki requirements.

## Supporting information


**Disclosure form**.

**Appendix S1:** Supplementary Information
