# Supplementary material for: Global Trends in Risk Factors for Low Back Pain: An Analysis of the Global Burden of Disease Study Data From 1990 to 2021
Source: Arthritis Care Res (Hoboken). 2025 Apr 3;77(7):837–47. doi: 10.1002/acr.25520 (PMC12209496; doi:10.1002/acr.25520)
Supplement: Supplementary file 2 — Appendix S1: Supplementary Information [file ACR-77-837-s002.docx]

**Global and regional trends in risk factors for low back pain. An analysis of the GBD study data from 1990 to 2021**

**Supplemental digital content**


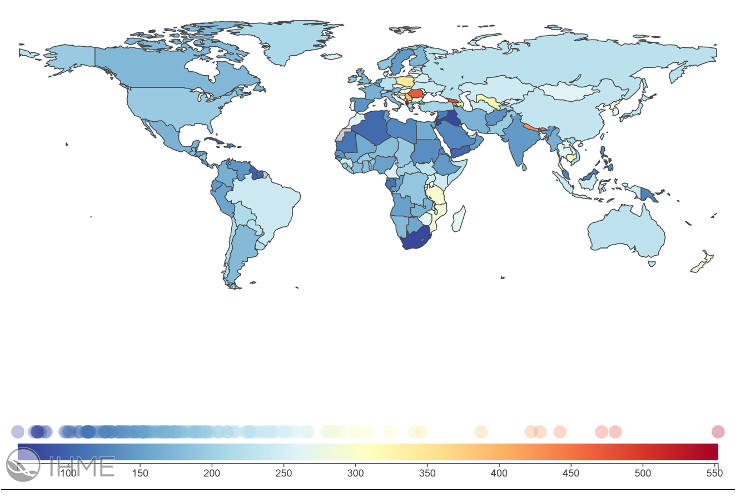


Figure S1: The number of YLDs due to low back pain attributable to occupational/ergonomic risk factors in 2021 presented for both sexes and all ages (from the GBD Compare tool) .


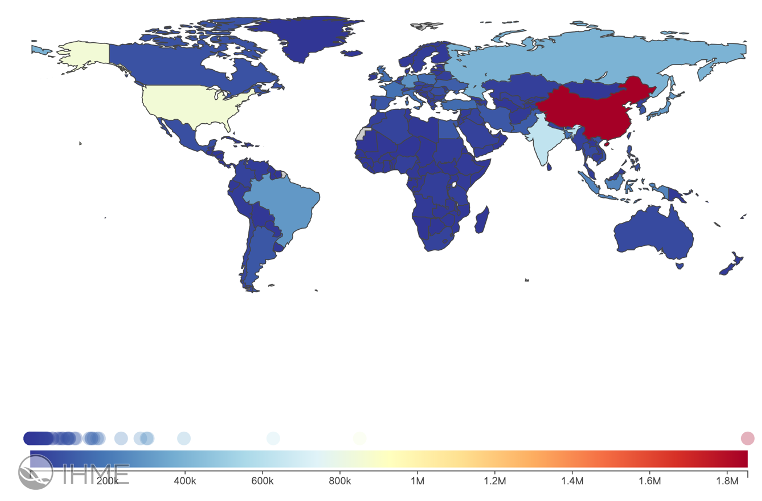


Figure S2: The number of YLDs due to low back pain attributable to behavioural risk factors (smoking) in 2021 presented for both sexes and all ages (from the GBD Compare tool).


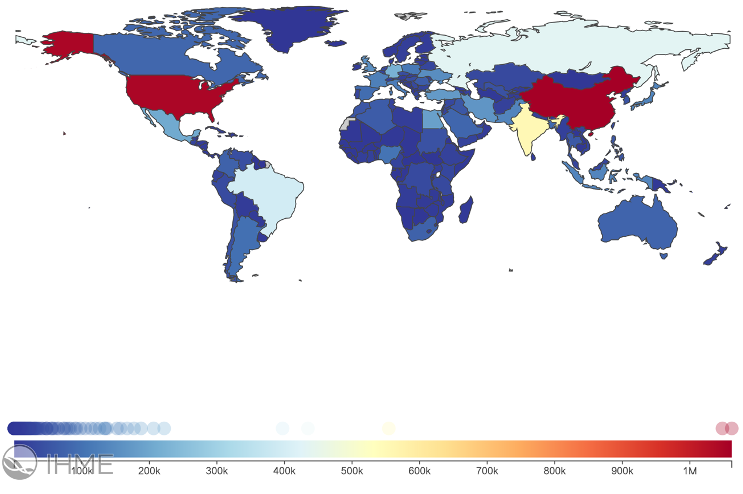


Figure S3: The number of YLDs due to low back pain attributable to metabolic risk factors (high BMI) in 2021 presented for both sexes and all ages (from the GBD Compare tool).

| Global, all ages | | | |
| --- | --- | --- | --- |
|  | Both sexes | Males | Females |
| 1990 | 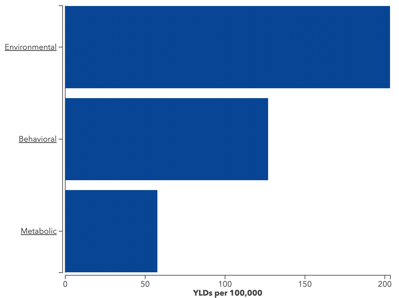 | 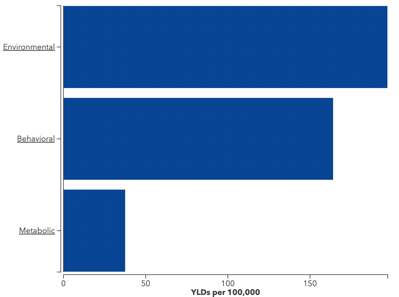 | 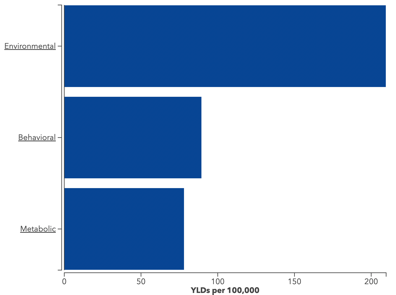 |
| 2021 | 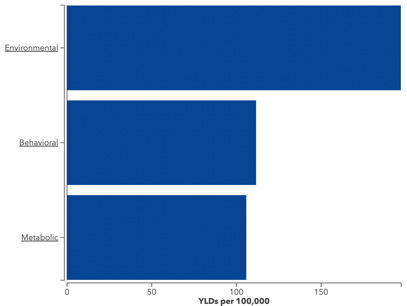 | 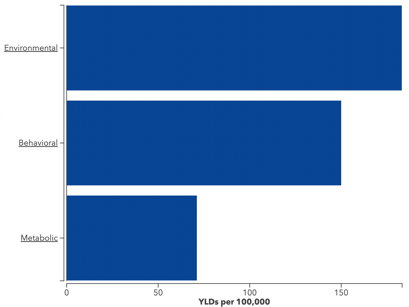 | 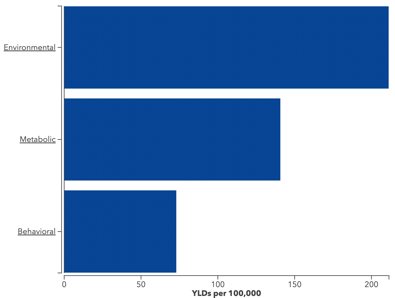 |
| High SDI areas, all ages | | | |
| 1990 | 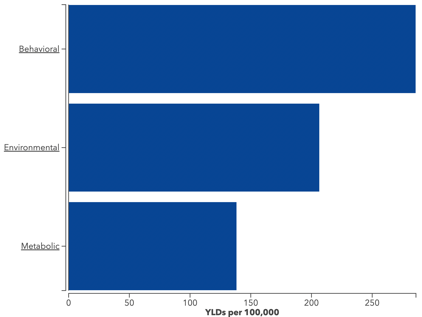 | 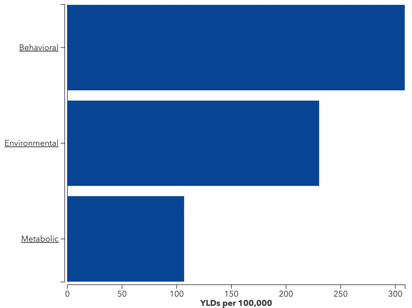 | 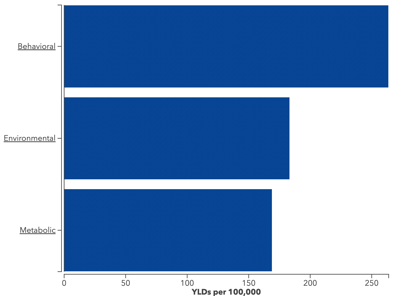 |
| 2021 | 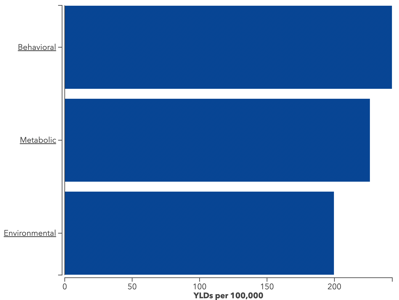 | 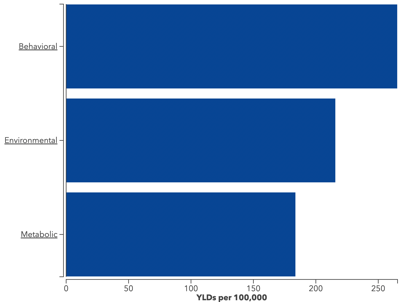 | 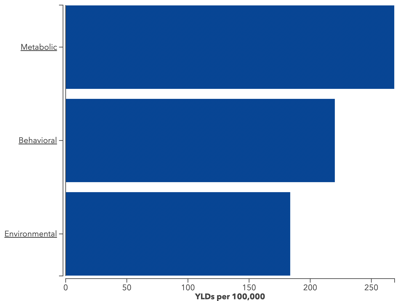 |
| Low SDI areas, all ages | | | |
| 1990 | 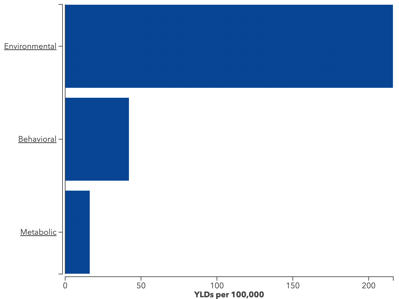 | 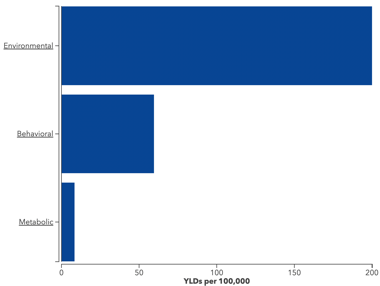 | 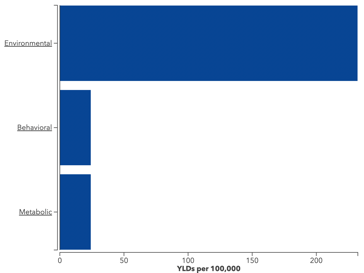 |
| 2021 | 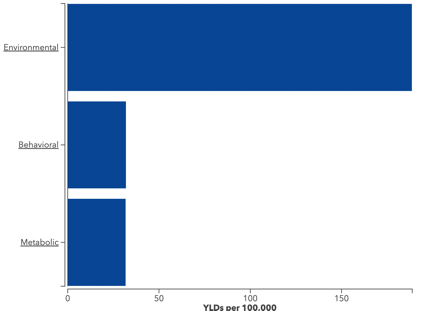 | 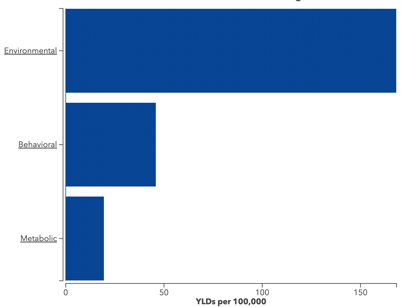 | 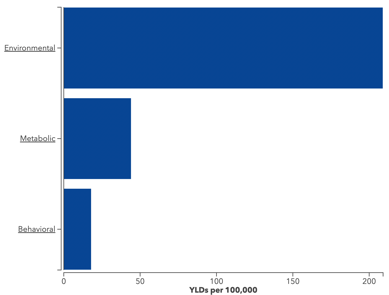 |

Figure S4: Rate of YLDs per 100,000 due to occupational ergonomic, behavioural (smoking) and metabolic (high BMI) risk factors in global, high SDI and low SDI areas in 1990 and 2021 presented for both sexes, males and females, of all GBD age groups

| Both sexes, all ages | Males, all ages | Females, all ages |
| --- | --- | --- |
| LBP attributable to occupational ergonomic risk factors | | |
| 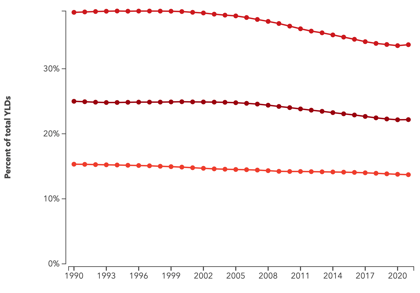 | 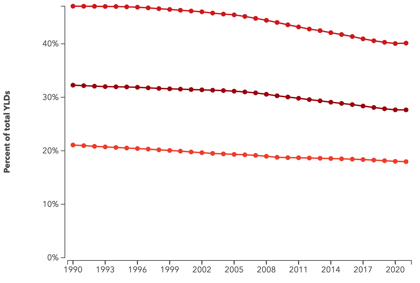 | 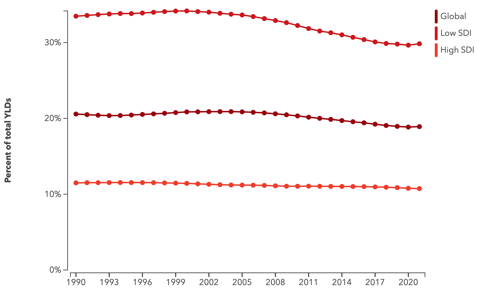 |
| LBP attributable to behavioural risk factors (smoking) | | |
| 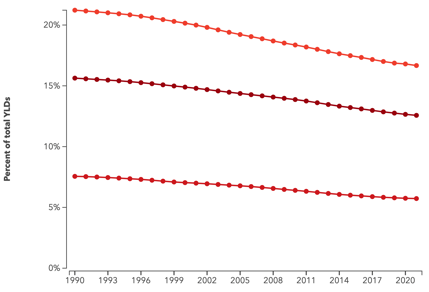 | 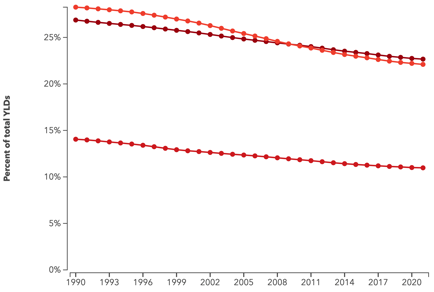 | 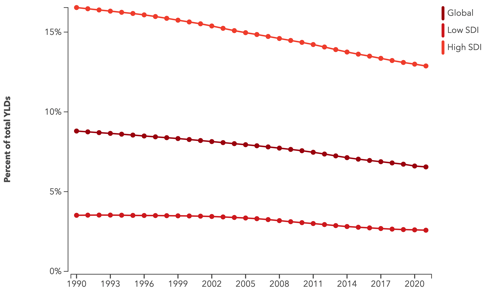 |
| LBP attributable to metabolic risk factors (High BMI) | | |
| 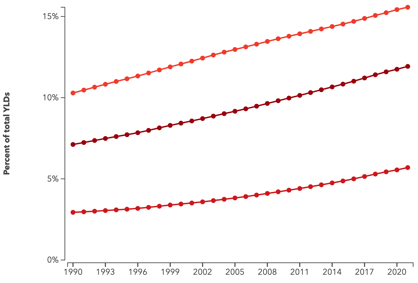 | 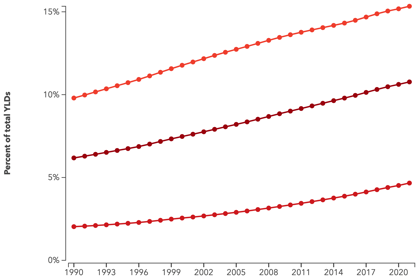 | 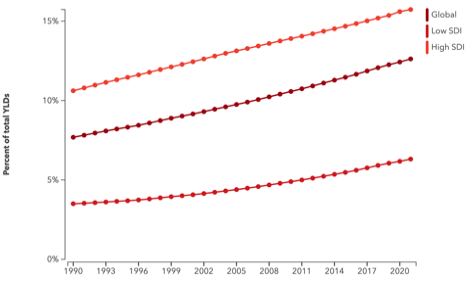 |

Figure S5: Comparison of trends in risk factors as a percentage of YLDs globally, in high SDI regions and low SDI regions from 1990 to 2021, for all GBD age groups.

Global, Females, All ages, Percent of total YLDs


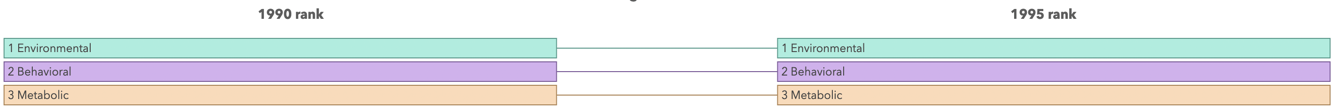


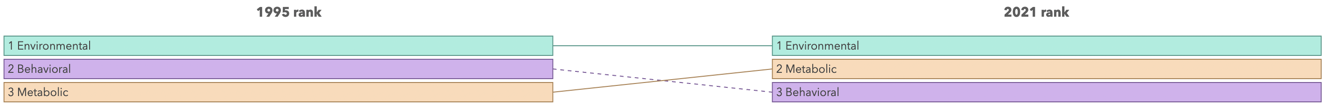


High SDI areas, Females, All ages, Percent of total YLDs


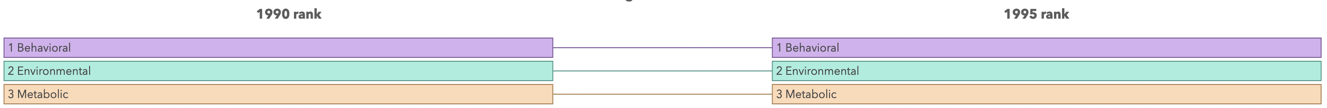


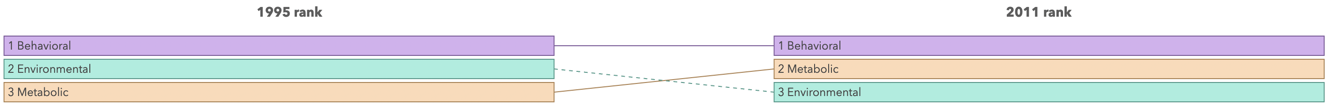


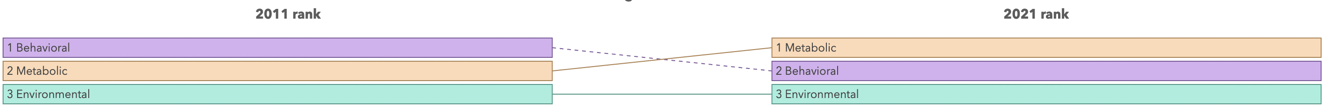


Low SDI areas, Females, All ages, Percent of total YLDs


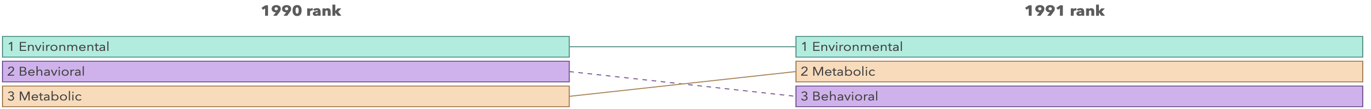


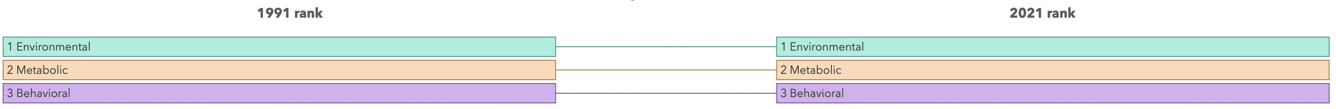


Figure S6. Rank of environmental (occupational ergonomic), behavioural (smoking) and metabolic (high BMI) risk factors as a rate of YLDs per 100,000 due to LBP in females, for all GBD age groups showing the year of ranking change.
